# The Complexities of “Minding the Gap”: Perceived Discrepancies Between Values and Behavior Affect Well-Being

**DOI:** 10.3389/fpsyg.2019.00736

**Published:** 2019-04-09

**Authors:** Megan Chrystal, Johannes A. Karl, Ronald Fischer

**Affiliations:** ^1^School of Psychology, Victoria University of Wellington, Wellington, New Zealand; ^2^Centre for Applied Cross-Cultural Research, School of Psychology, Victoria University of Wellington, Wellington, New Zealand; ^3^Instituto D'Or de Pesquisa e Ensino, São Paulo, Brazil

**Keywords:** values, behavior, well-being, self-determination theory, ACT

## Abstract

Research on self-determination theory and clinical models such as acceptance and commitment therapy has shown that behaving in line with our values is a key to maintaining healthy well-being. Combining work on values and experimental studies on moral hypocrisy and well-being, we experimentally tested how behaving incongruently with values affects well-being. We hypothesized that discrepancies between how one thinks one should have behaved and how one reported one did behave would be more detrimental to well-being when the behaviors were value-expressive and motivationally coherent compared to a control condition; greater perceived gaps between how participants feel they should have acted and how they report they did act would be associated with more negative well-being outcomes; the relationship between value manipulation and well-being would be mediated by perceived behavioral gap; and that personal values would interact with value manipulation to produce differential effects on well-being. One-hundred and fifty-eight first-year psychology students participated in an experiment designed to highlight discrepancies between how participants have behaved in accordance with a certain value and how they think they should have behaved, before reporting their well-being. As hypothesized, greater discrepancies between reported past behavior and how participants thought they should have behaved was associated with negative affect and decreased reports of positive well-being. We found no evidence for differential effects of manipulated value-expressive behaviors on well-being, or for our hypothesis that personal values and manipulated value-expressive behaviors interact. Nevertheless, value content mattered in terms of inducing perceived behavioral gaps. Our study suggests that perceived discrepancies between any value and reported past behavior can have a negative impact on some aspects of well-being. We discuss how the application of our methodology can be used in further studies to disentangle the value-behavior nexus.

## Introduction

Values are abstract concepts about ideal ways of living and end-states that act as guiding principles in people's lives (Schwartz, 1992). However, anyone who values kindness but has said something regrettable during an argument, or who values achievement but has procrastinated on work projects knows that though values may guide our behavior, we do not always act on our values. Furthermore, many people will be familiar with the negative feelings that may follow such transgressions. The relationship between values, behaviors, and well-being is the focus of this study. Through novel application of a method previously used in the study of religious hypocrisy (Yousaf and Gobet, 2013), we aim to investigate the consequences for people's well-being when they are made aware of the fact they have not acted according to specific values. Using an experimental method, we can explicitly test whether not acting in line with values can decrease well-being ratings temporally. Furthermore, it allows us to investigate whether some values are more strongly related to well-being, if value-congruent behaviors are experimentally reduced.

Schwartz (1992) has proposed a nearly universal theory of values in which values are organized in a circular structure based on their motivational compatibilities and conflicts. In this circle, compatible values are situated close to each other while conflicting values sit further away from each other. The values that make up this circle include up to 19 refined values that can be partitioned into 10 basic values or four higher-order values (Schwartz et al., 2012). The higher-order values are self-transcendence (caring for others both close and distant), openness to change (being motivated by seeking novel experiences), conservation (being motivated to maintain traditional order and stability), and self-enhancement (motivation to seek status, power and pursue socially accepted ways to distinguish oneself). Self-transcendence and self-enhancement values are opposed to each other and sit on opposite sides of the circle as self-transcendence emphasizes concern for the welfare of others and the environment, while self-enhancement emphasizes one's individual accomplishments and authority over others. Openness to change and conservation sit on opposite sides of a second orthogonal dimension where openness to change values prioritize independence, individual choice, and spontaneity while conservation values prioritize harmonious relationships, security, and tradition. While individuals share the same value structure, they differ in the importance they place on each value so that some people find openness to change values important guiding principles in their lives while others do not, for instance. Various different additional distinctions can be drawn into this two-dimensional space; for example, conservation values and self-transcendence values are socially-focused, while openness to change and self-enhancement values are more person-focused (see Schwartz et al., 2012; Fischer, 2017). Distinct values in this two-dimensional space have been found to relate to other variables, such as attitudes and behavior (Maio and Olson, 1995), social attitudes (Boer and Fischer, 2013), personality traits (Fischer and Boer, 2015), emotions (Higgins, 1987), and well-being (Sagiv and Schwartz, 2000, for a general overview of the relationships between values and behavior, see Roccas and Sagiv, 2017).

Self-determination theory (SDT, Deci and Ryan, 1985) provides one theoretical angle for unraveling how specific values influence well-being^1^. Deci and Ryan proposed that autonomy, relatedness, and competence are innate psychological needs. Satisfaction of these needs is inherently rewarding and key to experiencing well-being (Ryan and Deci, 2000). Conversely, the pursuit of more extrinsically reinforced motivations, such as financial success, do not provide direct satisfaction of innate needs and can ultimately diminish well-being (Kasser and Ryan, 1996). Kasser (2002) argued that Schwartz's (1992) values model converges with Kasser and Ryan's (1996) distinction between intrinsic and extrinsic motivations that underlies self-determination theory. Kasser (2002) suggested that stimulation and self-direction (openness to change) overlap with autonomy; benevolence and universalism (self-transcendence) with relatedness; and power and achievement (self-enhancement), as well as conformity/tradition (conservation), with extrinsic motivations such as pursuing financial success, and social recognition (for a similar conceptualization based on world-wide data, see also Fischer and Schwartz, 2011). This classification is not shared by all researchers for all values: for example, achievement may reflect the intrinsic motivator of competency (e.g., Bilsky and Schwartz, 1994) or achievement may be seen as either extrinsically or intrinsically motivating depending on individuals and their context (Bobowik et al., 2011). For example, one could be motivated to pursue achievement goals in order to gain social recognition (extrinsic) or to experience feelings of competence (intrinsic). These debates of competence-related values notwithstanding, the motivational circle described by Schwartz (1992) and the proposals from SDT have inspired research into how values may influence well-being.

In their seminal study, Sagiv and Schwartz (2000) correlated scores of 10 personal values with three aspects of subjective well-being to test the direct effect these values may have on well-being. In six samples from three different cultural groups, they found that achievement, stimulation, and self-direction were positively correlated with positive affect, while security, conformity, and tradition were weakly and negatively correlated with positive affect as they predicted. However, the predictions that benevolence and universalism would correlate positively, while power would correlate negatively with positive affect were not supported. Furthermore, none of the values correlated in any significant way with satisfaction with life. In a more recent study, Karabati and Cemalcilar (2010), performed correlations between the same 10 values and subjective well-being measures in a sample of Turkish students while controlling for materialism (the importance placed on worldly possessions). Contrary to Sagiv and Schwartz's (2000) results and what one would expect based on SDT, tradition, conformity, and security were weakly and positively correlated with subjective well-being while stimulation, Self-Direction, and universalism were weakly and negatively correlated with subjective well-being. These studies indicate that the relationships between specific values and well-being are weak and inconsistent across samples (see Schwartz and Sortheix, 2018, for a comprehensive review of this literature).

One reason for the inconsistent support for specific values' relations to well-being may be that this basic relationship is influenced by several moderating and mediating factors. In their second study, Sagiv and Schwartz (2000) considered how the way in which an individual's values align with those of the people around them may influence the relationship between values and well-being. They found that the more students valued power in an environment conducive of power (i.e., in a business school), the more general mental health and satisfaction with life they experienced. However, the more students valued power in an environment where power was not valued highly (i.e., in a psychology school), the less positive affect and satisfaction with life they experienced. Karabati and Cemalcilar (2010) suggested that their unexpected results may be due to Turkish society being a collectivist society in which conservation values are fostered while openness to change and some aspects of self-transcendence are not supported. Therefore, people who value the latter may experience alienation that negatively impacts well-being. Testing these relationships directly, Sortheix and Lönnqvist (2014a) addressed value congruence with student samples from Argentina, Bulgaria, and Finland. Although they found non-significant relationships between four higher-order values and subjective well-being, they did find that congruence between an individual's values and that of their peers was related to positive measures of subjective well-being, and that social relationships partially mediated this relationship. Other variables found to influence this relationship include socioeconomic development (Sortheix and Lönnqvist, 2014b), egalitarianism (Sortheix and Schwartz, 2017), and personality traits (Haslam et al., 2009). The relationship between values and well-being therefore appears complex and a number of variables are likely to play a role (see also Schwartz and Sortheix, 2018). The current study introduces the concept of value-expressive behavior to highlight the possible role of whether individuals feel that they have acted on important values as another factor influencing the relationship between values and well-being.

Value-expressive behavior, as defined by Bardi and Schwartz (2003), refers to behaviors that primarily express motivational content of one value (e.g., behavior that expresses benevolence). We aim to examine how perceptions that one has acted upon specific values–or not doing so–affects well-being. To illustrate, is valuing self-direction in itself still beneficial to well-being when one does not or cannot express this value by behaving in a self-directed manner?

Sheldon and Krieger (2014) investigated the impact that endorsing and behaving on intrinsic values such as personal growth (similar to achievement) relative to extrinsic values such as having status and fame (similar to power) had on measures of well-being. Participants indicated how important intrinsic and extrinsic values were to them and then reported to what extent they actually behaved in ways that expressed these values. Prioritization of intrinsic relative to extrinsic values significantly predicted lower depression and anxiety, and more positive affect. This relationship was moderated by value-expressive behavior for depression so that those high in intrinsic relative to extrinsic values who reported more behavior expressing these values experienced the least depression. The relationship was also moderated for positive affect so that those high in intrinsic relative to extrinsic values who reported more behavior expressing these values experienced the most positive affect. Moreover, participants whose personally important values and behavior were congruent had more meaning in life and searched less for meaning in life.

These empirical findings are mirrored in a number of clinical models and therapeutic approaches. Acceptance and commitment therapy (ACT), for example, includes valued behaviors as a central component to the therapeutic model (Hayes et al., 1999). The purpose of ACT is to help clients create a rich and meaningful life. Identifying personal values and taking committed action on these are critical steps for clients to achieve that goal, regardless of what those values actually are as long as they are important to the client (Harris, 2009). In this regard, ACT differs from SDT since any value is deemed important for well-being as long as the individual deems that value important for him or herself.

Clinical research has demonstrated the effectiveness of this approach. For example, Vowles and McCracken (2008) administered an intervention focusing on acceptance and value-expressive behaviors (relating to the value domains of family, intimate relations, friends, work, health, and growth/learning) to participants with chronic pain. They found that individuals who engaged in more value-expressive behaviors at follow-up 3 months later compared to pre-intervention experienced less pain-related distress, pain-related anxiety, and depression (among other outcomes). In summary, current research suggests that acting on one's values is more likely to be associated with well-being in a broad sense than either valuing some abstract goal alone without pursuing it further or performing some behavior without it being connected to some value. Different mechanisms might drive these effects, including feelings of competence and efficacy when acting on one's values (Bandura, 1977; Fischer, 2017), or reduced feelings of cognitive dissonance and increased feelings of perceived authenticity (Festinger, 1957; Goldman and Kernis, 2002). Yet, as reviewed by Schwartz and Sortheix (2018), some studies have found that value-expressive behaviors leading to goal attainment are not always associated with improved well-being if the values are extrinsic. Therefore, value content seems to matter. These different result patterns indicate a need for further research in this area.

We adapt and test a new method of examining the relationship between values, behavior, and well-being, but focusing on the effect that perceiving a discrepancy between how one has acted and how one should have acted on a value has on well-being. The method we use is an altered version of that used by Yousaf and Gobet (2013) in their study of religious hypocrisy. They asked participants first to write why certain religious (or neutral, in the control condition) behaviors are important, then how often the participants had performed these behaviors, and lastly how often they thought they should have performed the behaviors (this latter measure is termed the “behavioral gap”). The intervals of the scales on which participants indicated how often they performed the religious behaviors were intentionally stretched so that participants were likely to respond with answers on the lower end of the scale. By having participants give written endorsement of religious behaviors and then indicate that they had performed low levels of these behaviors relative to the levels at the higher end of the scale, this method was designed to induce feelings of hypocrisy. Their Experiment 2 found that people in the experimental group experienced greater guilt and shame, and general discomfort compared to controls (only guilt and shame were significantly greater in their experimental compared to control condition in their other two experiments, however). Our current study adapted this method to the study of value and self-reported behavior discrepancies and well-being by asking about value-expressive behaviors in four values conditions (self-enhancement, self-transcendence, openness to change, and conservation), and motivationally mixed behaviors in the control condition, and measuring aspects of well-being as the outcome variables. The study by Yousaf and Gobet (2013) focused on a single behavioral domain. The advantage of using the Schwartz theory is that it differentiates values in terms of their motivational orientation. For any individual, it will be difficult to pursue all values to a similar degree. This is also relevant for clinical applications because our study can provide novel insights into which values might be more relevant for well-being.

To test whether their manipulation worked, Yousaf and Gobet (2013) looked at the behavioral gap—the measurement of how much participants thought they should have acted—which ranged from a lot less to a lot more than what they did. By comparing the behavioral gap measurements of each experimental condition to the control, we will be able to determine if the manipulation has worked to increase behavioral gap above and beyond the effect of asking about neutral behaviors and responding on normal scales used in the control condition.

H1: If our method can induce perceived discrepancies between how participants acted on value-expressive behavior and how they think they should have acted, the perceived behavioral gap should be greater for each of the experimental conditions compared to the control.

Based on clinical theories on the importance of value-behavior congruence regardless of the content of values, we test the following hypothesis about the effect our manipulation may have on well-being:

H2: If behaving incongruently with values leads to decreased well-being, then inducing perceived discrepancies between expected and self-reported value-expressive behavior should lead to decreased well-being compared to the control condition.

We explore whether discrepancies between expected behavioral expression and self-reported behavior have differential effects on well-being depending on what value those behaviors express. As outlined above, values have different motivational orientations and previous research has suggested that values have differential relationships with well-being, therefore, behavioral expression of values may also affect well-being differentially. However, given the complex direct effects found in the literature as discussed above, we make no specific hypotheses on how exactly different value-expressive behaviors may affect well-being, but rather treat this aspect of our study as exploratory. These comparative findings of the specific motivational content of values in our study are informative for future research.

Our method is based on the premise that our manipulations will result in greater perceived behavioral gaps (participants feel that they should have displayed more of the value-expressive behaviors) compared to a control condition. This recognition of inaction—a feeling of “I should have acted more”—is then thought to affect well-being. Therefore, we predict that perceived behavioral gaps are associated with decreased well-being:

H3: Perceived behavioral gaps (i.e., recognition of one' own inaction) are negatively associated with positive well-being indices.

**Figure 1:**
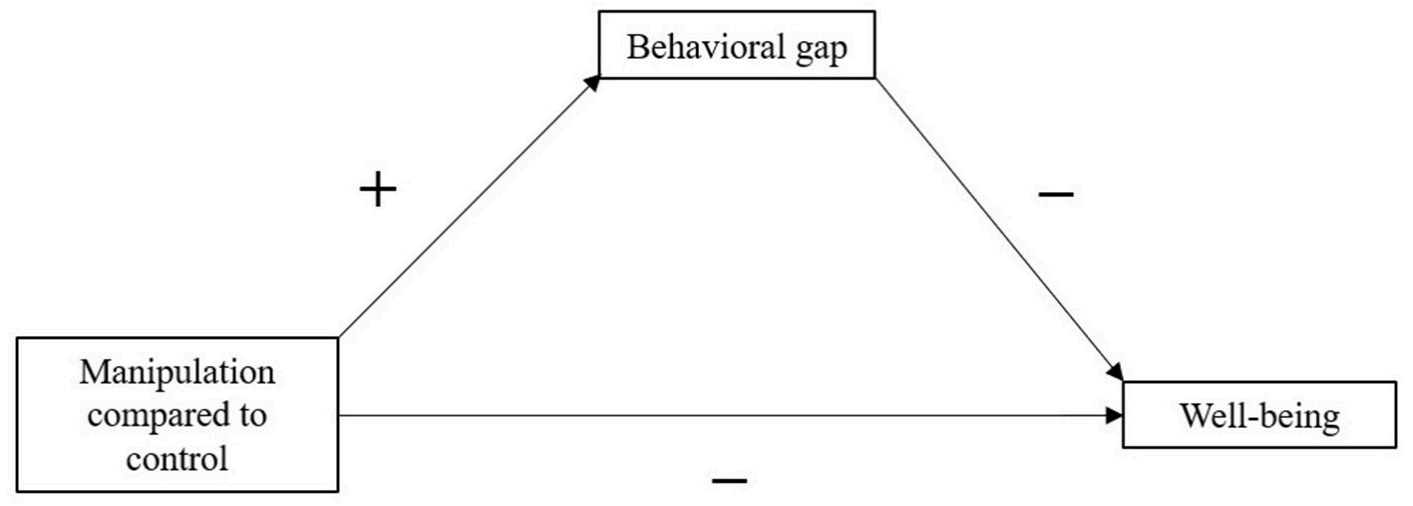
Proposed mediation model of the relationships between manipulations, behavioral gap, and measures of well-being.

To the extent that perceived behavioral gaps are the drivers of reduced well-being, we should expect that perceived behavioral gap explain the differences between the experimental and control condition. In other words, the relationship between experimental conditions and well-being is predicted to be mediated by this perceived behavioral gap in past value-expressive behavior (this mediation model is depicted in Figure 1).

H4: The relationship between experimental condition and well-being should be mediated by the perceived behavioral gap in value-expressive behavior.

The manipulations are not intended to manipulate participants' personal values directly, they are intended to generate perceptions that one should have performed more value-expressive behaviors relating to one of the four higher-order values. Participants will vary in the degree to which they personally prioritize the manipulated values in the condition they are in. The extent to which our manipulation works to induce discrepant perceptions of one's past behavior may be dependent on whether participants personally prioritize the specific value that was experimentally manipulated (it is a between-subject design). In other words, personal values are expected to moderate the relationship between the manipulated value condition and perceived behavioral gaps in value-expressive behavior. The experimental manipulation may be more effective if the values of the participant are aligned with the experimentally induced value-expressive behaviors.

H5: Individuals should feel as though they should have done more of certain value-expressive behaviors (i.e., have a greater perceived behavioral gap) when those behaviors express a value they find personally important, compared to a value they consider less important.

## Method

### Participants

Participants were 158 first-year psychology students from the Victoria University of Wellington who completed the study voluntarily for partial course credit. Ethical approval was given by the School of Psychology Human Ethics Committee under delegated authority of Victoria University of Wellington's Human Ethics Committee. We conducted a power analysis with G^*^Power 3.0.10 (Faul et al., 2007). The a priori test was based on the one-way ANOVA needed to check the manipulation worked (main effect of manipulation on the behavioral gap measure). With 5 conditions, an alpha rate of 0.05, expected power of 0.80, and an effect size of *f* = *0.3*0 (based on finding a medium to large effect; Cohen, 1992), this analysis suggested a necessary sample size of 140.

## Materials

### Value-Behavior Gap Manipulation

We manipulated the salience of value and reported past behavior discrepancies by adapting a procedure developed by Yousaf and Gobet (2013). The manipulation consisted of three phases, the first two of which were intended to increase participants' feeling that they did not act in line with values and the third was designed to measure the extent to which individuals felt they should have acted in line with the manipulated values. We use the term “perceived behavioral gap” for the measures taken at the third phase of the experiment (relevant for testing H3 and H4).

In the first phase, participants were asked to complete sentences that prompted them to give reasons for why six behaviors associated with one of the four higher-order values dimensions are important for people in general to do. For example, one of the behaviors participants in the self-transcendence condition were prompted with was “It is important for people to buy environmentally friendly products because…” This phase was intended to highlight the importance of these behaviors and why one should do them. These behaviors were taken from the Everyday Behavior Questionnaire developed by Schwartz and Butenko (2014) and some were modified to be more relevant to our sample of New Zealand university students. The face validity of each behavior was evaluated by the authors before being used in the study. For participants in the control condition, the six behavior prompts were randomly selected from all possible higher-order value behaviors. That is, participants in the control condition were asked to write reasons for the importance of a range of behaviors related to self-enhancement, self-transcendence, conservation, and openness to change. This was done so that the control differed from the value condition manipulation as little as possible. We discussed a number of options for selecting behaviors that are value neutral, but after extended discussions in our lab group, we were unable to identify clearly neutral behaviors that did not have some motivational value-related content. Hence, we decided to use motivationally mixed behaviors. All participants were required to write at least 140 characters to complete each sentence to ensure sufficient time was spent considering the importance of each behavior.

During the second phase, we asked participants to indicate to what extent they had engaged in each of the six behaviors they had written about in the first phase. The time frame put on each past behavior was dependent on how often the behavior was likely to take place, based on discussion amongst our laboratory group. For example, in the conservation condition, participants were asked “How many times have you changed your passwords in the last year?,” whereas in the openness to change condition, participants were asked “How many times have you treated yourself in the last 7 days?” because this behavior is likely to be more regularly engaged in than the former. Therefore, our manipulation took into account prototypical opportunities to engage in a particular behavior. We did not specifically ask participants how often they had done the behaviors in relation to the opportunities they had to do them. Therefore, the 9-point scale for each behavior was intentionally stretched so that most participants would need to answer on the lower end of the scale, highlighting how little they had engaged in the behaviors they had discussed the importance of in phase 1. For example, those asked about how often they had changed their passwords in the last year were presented with the following scale: *None at all, 1 time, 2 times, 3-5 times, 6-8 times, 9-11 times, 12-14 times, 15-17 times, 18*+ *times*. In the control condition, participants were asked about six behaviors they had not been asked about in the first phase and, importantly, responded to scales that were not stretched. Therefore, answering these questions was intended to not highlight discrepancies between intended and reported value-expressive behavior (because the behaviors from phase 1 and 2 were not matched and the response scale was not artificially stretched). For example, participants responded to “How often have you chatted to someone before a lecture in the last 7 days?” on the following scale: *None at all, 1–2 times, 3–4 times, 5–6 times, 7–8 times, 9–10 times, 11–12 times, 13–14 times, 15*+ *times*. These frequencies were discussed with students to get reasonable base rates. Hence, this control condition was intended to leave participants feeling neutral about their behavior as they were not given high expectations for their behavior nor were the behaviors matched to the values that participants had seen before.

During the final third experimental phase, we measured the gap between how participants behaved and how they think they should have behaved (perceived behavioral gaps). To the extent that participants felt that they should have performed more value-expressive behaviors, we expect that these participants will experience lower well-being (see our H3). All participants in all conditions were asked to what extent they thought they should have engaged in each of the six behaviors they were previously asked about at phase 3. Importantly, these behaviors matched between phases 1, 2, and 3 for the experimental conditions, but were not matched for the control condition. Answers were indicated on a 9-point scale from−4 *A lot less often/money/time than I did* to 4 *A lot more often/money/time than I did*. The midpoint, 0, was *the same amount that I did*. All questions and scales included in the manipulation surveys can be found in Appendix A of the Supplementary Material.

### Well-Being Variables

We aimed to use a wide range of well-being measures to capture both subjective and psychological aspects of well-being (Keyes et al., 2002).

#### Positive and Negative Affect

Positive and negative affect was measured with the Positive and Negative Affect Schedule (PANAS; Watson et al., 1988). The PANAS uses 10 items, such as “inspired,” to measure positive affect and 10 items, such as “irritable” to measure negative affect. Participants were asked to indicate on a five-point scale from 1 *very slightly or not at all* to 5 *extremely* to what extent they felt each emotion at the present moment. Scores for each subscale were averaged to create overall scores for both positive and negative affect. We added five extra negative emotions to the PANAS to capture a more comprehensive set of affect in line with previous studies (e.g., Yousaf and Gobet, 2013): disappointed, frustrated, embarrassed, sad, and anxious. Internal reliability of the positive and the combined negative affect scale were excellent with a Cronbach's alpha of 0.89 and 0.91, respectively (removing the additional negative affect items decreased alpha: α = 0.87).

#### Meaning in Life

The Meaning in Life Questionnaire (Steger et al., 2006) was used to measure both the presence of meaning in life and searching for meaning in life. According to Steger et al. (2009), the presence of meaning in life is generally considered an aspect of psychological well-being, while searching for meaning in life is associated with negative well-being outcomes. The five items for the presence of meaning in life include items such as “I understand my life's meaning” and an example of one of the five items for search for meaning in life is “I am searching for meaning in life.” Items were rated on a seven-point scale from 1 *absolutely untrue* to 7 *absolutely true*. After reverse coding scores for negatively phrased items, average scores for each subscale were calculated to create an overall score for each subscale. Internal reliability for presence of meaning in life was excellent with Cronbach's alpha = 0.89, and similarly reliable for search for meaning in life with Cronbach's alpha = 0.88.

#### Life Satisfaction

Life satisfaction was measured with Diener et al. (1985) Satisfaction with Life Scale. This five-item measure includes items such as “In most ways my life is close to ideal” rated on a scale from 1 *strongly disagree* to 7 *strongly agree* and total scores are calculated by averaging the scores for each item. This measure had excellent internal reliability in this study with Cronbach's alpha = 0.87.

#### Resilience

The Brief Resilience Scale (Smith et al., 2008) is a six-item measure designed to measure one's ability to recover from stressful situations. Items such as “I tend to bounce back quickly after hard times” were rated on a five-point scale from 1, *strongly disagree* to 5, *strongly agree*. After reverse coding reversed items, an average resiliency score is calculated. Internal reliability of the scale in the current study was excellent at α = 0.87.

#### Self-Esteem

The Rosenberg Self-Esteem Scale (Rosenberg, 1965) was used to measure self-esteem. Participants rated 10 items, such as “On the whole, I am satisfied with myself” on a four-point scale from 1 *strongly disagree*, to 4 *strongly agree*. After reverse coding reversed items, a total score of self-esteem was calculated by averaging item scores. This scale had excellent internal reliability in this study with α = 0.91.

### Personal Values

We measured individuals' personal values with a shortened version of the revised Portrait Values Questionnaire (PVQ-RR; Schwartz et al., 2012). The PVQ-RR has a total of 57 items with three items measuring each of the 19 values facets. Items described a hypothetical person and participants then rate how similar this person is to them from 1 *Not like me at all* to 6 *Very much like me*. An example of an item that measures power resources is “It is important to him/her to be wealthy.” To keep the survey brief, we used one item to measure each of these 19 values facets, which were chosen at random (see Appendix C of the Supplementary Material for the list of chosen values).

We combined means of universalism-nature, universalism-concern, universalism-tolerance, benevolence-care, and benevolence-dependability items to create a score for self-transcendence; means for achievement, power dominance, and power resources to create a score for self-enhancement; means for self-direction thought, self-direction action, stimulation, and hedonism items to create a score for openness to change; and combined means for security-personal, security-societal, tradition, conformity-rules, conformity-interpersonal, humility, and face value items to create a score for conservation. The internal reliability of the self-transcendence, openness to change, and conservation subscales in the PVQ-RR were acceptable at α = 0.81, 0.74, and 0.78 respectively. Self-enhancement, however, was less reliable at α = 0.66, possibly due to having the lowest number of items (three) out of the value subscales. However, it is noteworthy that our lowest reliability score was still within the medium reliability reported for value instruments (see Schwartz and Rubel, 2005). Scores were not ipsatized as recommended by Schwartz (2016) when values are used as predictors in a regression model, as we planned to do for H3.

### Procedure

Participants signed up to take this survey via the survey program Qualtrics and, after giving informed consent, were randomly assigned to either the self-transcendence (*n* = 29), self-enhancement (*n* = 32), openness to change (*n* = 34), conservation (*n* = 33), or control (*n* = 30) condition. To summarize the experimental procedure, the conditions varied the types of behaviors that participants were asked about. Hence, one condition focused on behaviors that express self-transcendence (the self-transcendence condition), another on behaviors that express conservation (the conservation condition), and so on. We manipulated behaviors expressing the four major value types and we also had a control condition, making five total conditions. The experimental vs. control conditions also varied on whether the scales in the second phase were stretched (in the experimental condition) or not (in the control condition). Finally, half of participants were presented with the PVQ-RR before the manipulation and half saw it afterwards to counterbalance any effects responding to personal values might have on subsequent answers about self-reported behavior, or any effects the manipulation may have had on the reporting of personal values. Linear model analyses including the order of the PVQ-RR completion did not show an effect of order on our dependent variables or personal values and, therefore, we do not further discuss this counterbalancing condition. After the manipulation, participants responded to the main dependent variables (measures of different aspects of well-being), as well as the Guilt and Shame Proneness scale (Cohen et al., 2011) that was not relevant for the current study. See Appendix B of the Supplementary Material for a table outlining the experimental procedure.

### Dimension Reduction of Well-Being Variables

The Pearson's correlations between each of the well-being measures were calculated to see relations between variables (see Table 1).

**Table 1:** Pearson's correlations between measures of well-being.

**Measure**	**1**	**2**	**3**	**4**	**5**	**6**	**7**	**8**
Presence of meaning in life	—							
Searching for meaning in life	−0.21^**^	—						
Negative affect	−0.18^**^	0.17^**^	—					
Positive affect	0.18^**^	0.09	0.18^**^	—				
Life satisfaction	0.55^***^	−0.13	−0.30^***^	0.21^**^	—			
Resilience	0.17^**^	−0.25^***^	−0.25^***^	0.21^**^	0.32^***^	—		
Self-esteem	0.51^***^	−0.41^***^	−0.41^***^	0.25^***^	0.61^***^	0.45^***^	—	
Behavioral gap	−0.03	0.12	0.18^*^	−0.09	−0.11	−0.21^**^	−0.21^**^	—

* *p < 0.05*,

** *p < 0.01*,

*** *p < 0.001*.

Because there were many significant relationships of various strengths, a principle components analysis was conducted to see how the various measures of well-being grouped together (and therefore reduce the number of outcome measures and Type I error). Scale scores based on the outcomes of the PCA were used as outcomes in the analysis. Parallel analysis suggested retaining two components, Eigenvalues = 2.73 and 1.07. Life satisfaction, having meaning in life, resilience, and self-esteem loaded onto Component 1; searching for meaning in life, and negative affect loaded onto Component 2. Positive affect loaded on both components, with a slightly stronger loading on Component 2. The first component can be interpreted in a straightforward fashion as measuring general positive well-being. The second component is less clearly interpretable, partly because the two affect measures and Meaning in Life Searching all loaded positively (probably suggesting that experiencing any kind of affect independent of valence correlates positively with searching for meaning in life, akin to a general emotionality factor). Because positive affect loaded positively onto Component 1, we included positive affect in this component and called it Positive Well-being. We analyze negative affect and searching for meaning in life separately in our analysis because we were interested in the specific effects given the complexity of this factor. In summary, we retained the composite scale measuring Positive Well-being, and the single scales of Negative Affect, and Meaning in Life Searching as outcome variables. Table 2 below shows results from our principal component analysis.

**Table 2:** Principle component analysis.

	**Component 1**	**Component 2**
Self-esteem	**0.84**	−0.23
Life satisfaction	**0.80**	−0.10
Presence of meaning in life	**0.72**	−0.05
Resilience	**0.57**	−0.20
Positive affect	**0.52**	**0.69**
Negative affect	−0.32	**0.70**
Meaning in life searching	−0.24	**0.56**
Eigenvalue	2.42	1.08
Variance explained	0.37	0.20

*Loadings above 0.4 are bolded*.

### Planned Analyses

#### Induction of Behavioral Gaps in Experimental Conditions: Hypothesis 1

We will use a one-way between-subjects ANOVA with experimental condition as the predictor variable and behavioral gap as the outcome variable to see if there is a main effect of experimental condition on behavioral gap. We will use an ANOVA for this analysis as the predictor variable is categorical and has more than two levels. The comparisons of interest are between each experimental condition and the control, so we will use planned comparisons to focus on these potential differences.

#### Effect of Perceived Discrepancies Between Expected And Reported Value-Expressive vs. Controls on Well-Being: Hypothesis 2

We will conduct Welch's two sample *t*-tests to see if there are differences in the mean scores of Positive Well-being, Negative Affect, and Meaning in Life Searching between those in any of the experimental condition vs. the control group. Welch's independent *t*-tests are appropriate as they do not assume equal sample sizes and because we are interested in simple mean comparisons between two groups (controls and those in any experimental condition).

#### Differential Value-Expressive Behavior Effects on Well-Being: Exploratory Analyses

For our exploratory analyses, we will use MANOVA with each value-expressive behavior condition as a predictor variable and the well-being variables as outcome variables. We will use MANOVA because the predictor variable is categorical with more than two conditions and there are multiple continuous outcome variables. If the overall MANOVA result is significant, *post-hoc* analyses will be conducted to identify where any significant differences in the effects of condition on well-being occurred.

#### Perceived Behavioral Gap Predicting Well-Being: Hypothesis 3

We will conduct three linear model regressions with perceived behavioral gap predicting Positive Well-being, Negative Affect, and Meaning in Life Searching to test H3. We will use regressions because we are interested in how behavioral gap affects these outcome variables (rather than if they are correlated) and because the predictor variable is continuous. We predict that the greater the perceived behavioral gap, the less Positive Well-being, and the more Negative Affect and Meaning in Life Searching participants will report.

#### Perceived Behavioral Gap Mediates the Effect of the Manipulation on Well-Being: Hypothesis 4

Three linear model regressions with the well-being outcomes, Positive Well-being, Negative Affect, and Meaning in Life Searching regressed onto experimental vs. control condition and perceived behavioral gap will be conducted. This method will allow us to see if behavioral gap has a mediating effect on the relationship between condition and each well-being outcome. The expectation is that the perceived behavioral gap, but not the experimental condition explains variance in the well-being variables.

#### Personal Values and Value-Expressive Behavior Manipulations Interact With Each Other: Hypothesis 5

To test the prediction that perceived behavioral gaps will be greatest when people personally prioritize the value of the condition they are in, perceived behavioral gaps will be regressed onto the interaction between each value condition and PVQ-RR scores for each value subscale. This method is appropriate as it will show how the interaction between value condition and PVQ-RR scores may affect behavioral gap scores.

#### Statistical Analysis Software

All analyses will be completed in R 3.4.3 (R Core Team, 2017) using RStudio version 1.1.423 (RStudio Team, 2016) with lavaan version 0.5-23.1097 (Rosseel, 2012), car version 2.1-6 (Fox and Weisberg, 2011), lsr version 0.5 (Navarro, 2015), heplots version 1.3-5 (Fox et al., 2018), and psych version 1.7.8 (Revelle, 2017) packages.

## Results

### PVQ-RR Scores

Average PVQ-RR scores for each of the four values were calculated to better understand the value profile of the sample. Self-transcendence was the most endorsed value (*M* = 5.13, *SD* = 0.71), followed by openness to change (*M* = 4.76, *SD* = 0.76), conservation (*M* = 4.31, *SD* = 0.75), and self-enhancement (*M* = 3.63, *SD* = 0.90).

### Testing Hypothesis 1: Effect of Conditions on Behavioral Gaps

We analyzed the perceived behavioral gaps to ensure the manipulation had the desired effect and did indeed lead to larger perceived behavioral gaps in the experimental conditions compared to the control group. We hypothesized that the behavioral gap measures should be greater for each of the experimental conditions compared to control.

A one-way between-subjects ANOVA was conducted to see if perceived behavioral gaps differed significantly between the experimental conditions and the control group. There was a significant main effect of manipulation condition on perceived behavioral gap, *F*_(4, 153)_ = 7.93, *p* < 0.001, η^2^ = 0.17. Key to our hypothesis are the comparisons between the control group's perceived behavioral gaps to each of the manipulation groups, therefore, we conducted planned comparisons. The mean perceived behavioral gap in the self-transcendence condition (*M* = 1.09, *SD* = 1.04) was significantly greater than those in the control (*M* = 0.58, *SD* = 0.72), *F*_(1, 153)_ = 10.65, *p* = 0.001. Perceived behavioral gaps in the openness to change condition (*M* = 1.10, *SD* = 0.82) were also greater than those in control, *F*_(1, 153)_ = 7.20, *p* = 0.008. The mean perceived behavioral gap in the self-enhancement condition, however, was significantly less than that of the control (*M* = 0.18, *SD* = 0.73), *F*_(1, 153)_ = 11.12, *p* = 0.001. Though the means were larger for the conservation value condition (*M* = 0.91, *SD* = 0.58) compared to the control (*M* = 0.58, *SD* = 0.72), this difference was not statistically significant, *F*_(1, 153)_ = 2.75, *p* = 0.10. Therefore, our H1 was only supported for the self-transcendence and openness to change conditions. Our data do not indicate that the remaining two conditions resulted in participants feeling as though they should have done more behaviors than those in the control condition^2^.

### Testing Hypotheses 2: Effect of Experimental Manipulation on Well-being

We performed Welch's independent *t*-tests to test whether manipulation of a behavioral gap compared to a control condition would result in lower well-being scores. There was no significant difference between scores for Positive Well-being for the control group (*M* = 3.49, *SD* = 0.54) and those in the experimental conditions (*M* = 3.35, *SD* = 0.69); *t*_(53.45)_ = 1.20, *p* = 0.23, *d* = 0.21. The effect size was small and direction of the differences was nevertheless in the predicted direction. There was no significant difference between scores of Negative Affect for the control group (*M* = 1.79, *SD* = 0.70) and those in the experimental conditions (*M* = 1.75, *SD* = 0.70); *t*_(43.85)_ = 0.31, *p* = 0.76, *d* = 0.06. Finally, scores for Meaning in Life Searching were significantly different between controls (*M* = 4.05, *SD* = 0.1.27) and those in the experimental conditions (*M* = 4.69, *SD* = 1.23); *t*_(42.60)_ = −2.52, *p* = 0.02, *d* = 0.52, showing that those in the experimental conditions reported more searching for meaning in life than did those in the control condition. Because the conservation and self-enhancement manipulations were shown not to work as intended, the analyses were re-run with these conditions excluded and this did not change the pattern of results. Overall, we did not find strong support for our Hypothesis 2.

### Exploratory Analysis: The Differential Effect of Experimental Conditions on Well-Being

To explore the differential effect of value-expressive behavior experimental conditions on well-being, we ran a one-way MANOVA with value-expressive behavior experimental condition as the independent variable and Positive Well-being, Negative Affect, and Meaning in Life Searching as dependent variables. A main effect of experimental condition on measures of well-being was approaching significance, Pillais' Trace = 0.13, *F*_(12, 459)_ = 1.70, *p* = 0.06, η^2^ = 0.043^3^.

**Figure 2:**
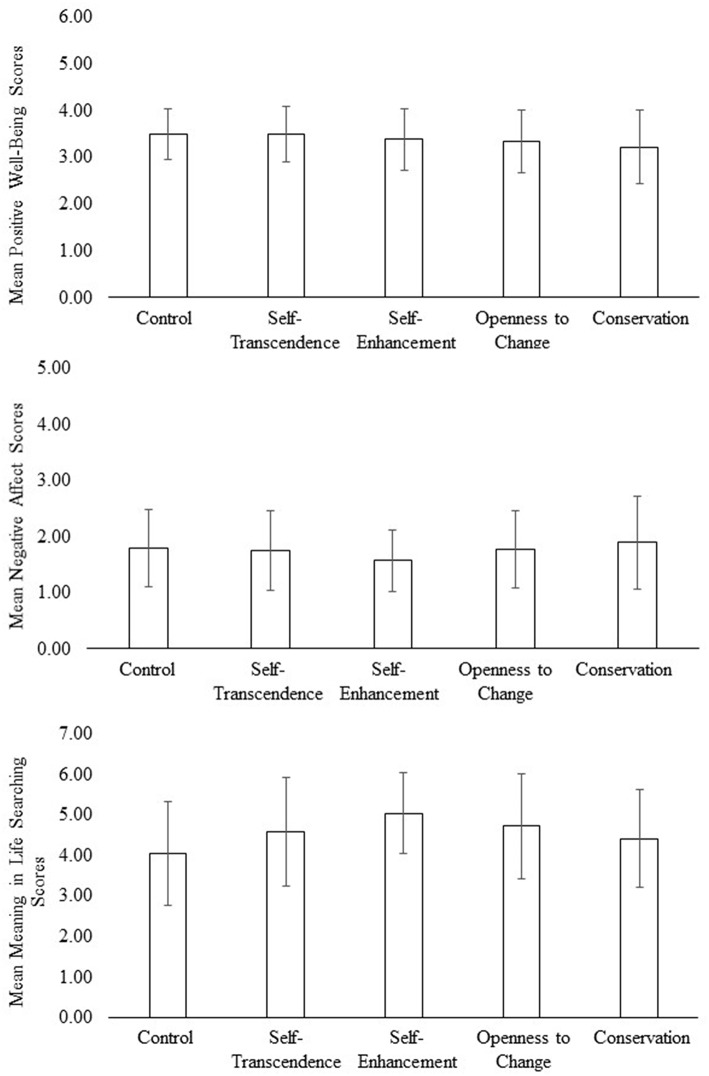
Mean scores of Positive Well-being, Negative Affect, and Meaning in Life Searching across each of the experimental conditions.

To follow up on these patterns (see Figure 2), we conducted three exploratory ANOVAs, one for each outcome variable. None of these ANOVAs were significant according to traditional levels of significance.

### Testing Hypothesis 3: Perceived Behavioral Gap Predicting Well-being

We performed regressions to test whether greater perceived behavioral gaps negatively affected well-being. We predicted that greater perceived behavioral gaps would be associated with less Positive Well-being and more Negative Affect and Meaning in Life Searching. Our first regression showed that greater perceived behavioral gaps were indeed associated with lower Positive Well-being, *B* = −0.13, *t*_(156)_ = −2.05, *p* = 0.04, *R*^2^ = 0.02. As expected, our second regression showed that greater perceived behavioral gaps overall were associated with greater Negative Affect, *B* = 0.15, *t*_(156)_ = 2.31, *p* = 0.02, *R*^2^ = 0.03. Our last regression, however, showed that perceived behavioral gaps did not have a significant relationship with Meaning in Life Searching, *B* = 0.17, *t*_(156)_ = 1.47, *p* = 0.14, *R*^2^ = 0.01. Overall, our H3 was supported for Positive Well-being and Negative Affect only.

### Testing Hypotheses 4: Perceived Behavioral Gap Mediates the Effect of Experimental Conditions on Well-being

**Figure 3:**
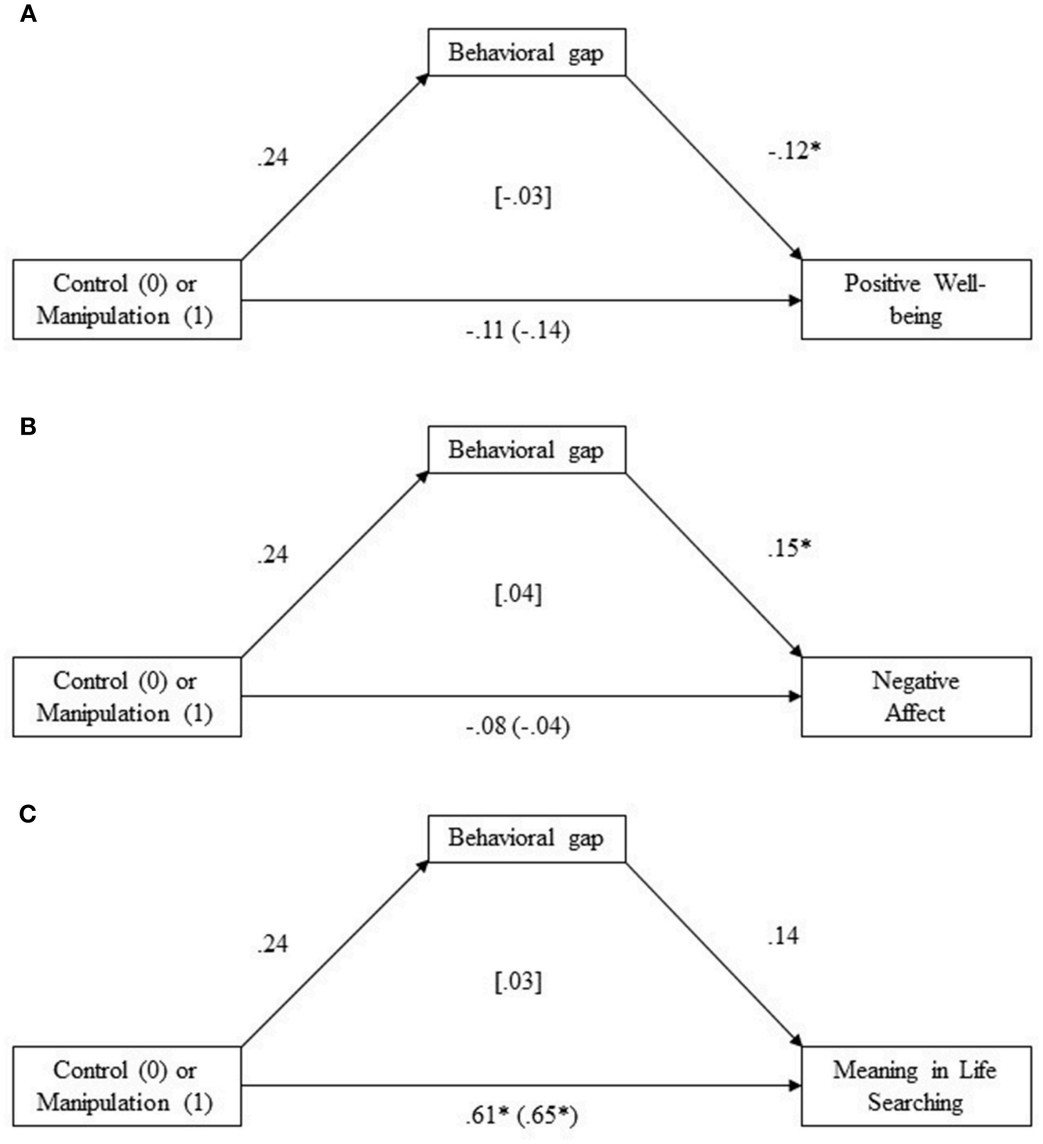
Unstandardized regression coefficients for the relationships between the presence of a manipulation, perceived behavioral gap, and **(A)** Positive Well-being, **(B)** Negative Affect, and **(C)** Meaning in Life Searching. The indirect effect is presented in square brackets; the total effect is presented in round brackets. *p < 0.05.

As reported in our tests for H2, there were no significant differences between the experimental and control groups for Positive Well-being and Negative Affect. Nevertheless, in accordance with recent recommendations indicating that indirect effects can be found in the absence of total and direct effects (Rucker et al., 2011) we tested for indirect effects of manipulation on all well-being measures by conducting mediations with the control (coded as 0) vs. experimental condition (coded as 1) as a predictor, perceived behavioral gap as a mediator, and each well-being measure outcomes. The indirect effects were tested using a bootstrap estimation method with 1,000 iterations. We ran three analyses, separately for each dependent variable. All indirect effects were non-significant, indicating that statistically significant mediations were not found in our sample. Overall results of the bootstrapped mediation analysis are presented in Figure 3. Therefore, our H4 (behavioral gap mediates the relationship between condition and well-being) was not supported.

### Testing Hypothesis 5: Personal Values' and Values Conditions' Effect on Perceived Behavioral Gap

To test whether value manipulations had a larger effect on perceived behavioral gap when the value was personally important to participants, regressions were performed in which the perceived behavioral gap was regressed onto the interaction between each condition with scores from each subscale of the PVQ-RR. Value conditions were dummy coded and compared to the control condition. Conditions and personal values together explained a significant portion of the variance among perceived behavioral gap scores, *F*_(24, 133)_ = 1.93, *p* = 0.01, adjusted *R*^2^ = 0.12. However, none of the individual regression coefficients were significant, with the minimum *p*-value for the main and interaction effects being *p* = 0.15. Our H5 was not supported because one's personal values and the value condition a person was assigned to did not interact to produce unique effects on the perceived behavioral gap. Similar regressions were performed for each well-being outcome which also showed non-significant interaction results. These results can be found in the regression tables in Appendix D of the Supplementary Material.

## Discussion

We used an experimental design as well as correlational analyses to examine the relationship between values, behavior, and well-being. To do this, we adapted a paradigm previously used in the study of religious hypocrisy to manipulate to what extent participants felt they should have done more or less of the behaviors expressive of a certain value. We measured a comprehensive number of well-being aspects to see the effect our manipulation and resulting perceived behavioral gaps had on these outcomes. Our results show that while our manipulation influenced perceived behavioral gaps for some values and perceived behavioral gaps seem to negatively affect well-being, the relationship between values, behavior, and well-being appears more complex.

Our first hypothesis concerned whether the method used by Yousaf and Gobet (2013) could induce greater perceived discrepancies between ideal and reported value-expressive behaviors compared to controls as it did in their studies using religious behaviors. We found that manipulations of self-transcendence and openness to change values increased perceptions of behavioral gaps compared to a control condition. However, there was no significant difference between conservation and control behavioral gaps (even though it was in the expected direction). Furthermore, the perceived behavioral gap for self-enhancement relevant behaviors was significantly smaller than controls. This suggests that value content matters. The different motivational content of the values affected the extent to which participants felt they should have done behaviors.

One might expect that because self-enhancement and conservation were the least important values for our participants based on their responses to the PVQ-RR (Schwartz et al., 2012), they may have been less motivated personally to perform these behaviors compared to those they found more important. A formal test of this was done in the context of Hypothesis 4. However, the interaction between PVQ-RR scores and condition did not significantly affect behavioral gap scores. This suggests that personally salient values did not interact with the manipulations. Hence, normatively salient values seem to induce perceptions of behavioral gap, but personally salient values do not. How can we reconcile these conflicting findings?

Our manipulation may have activated social norms around value content. We asked individuals to provide reasons of why certain (value-related) behaviors are important for people to do. This may have tapped into normatively sanctioned scripts and perceptions of moral obligations that are relatively shared within a cultural community (see also Fischer, 2006, for a related approach to measure normative perceptions of cultural values). It may be plausible that only those values that are salient within a population (but not necessarily salient for an individual) can activate feelings of behavioral gaps (e.g., not performing normatively salient behaviors). In the same vein, behaviors that are not normatively salient or are counter-normative (e.g., self-enhancement values) in a population, can decrease perceptions of behavioral gap. These findings are interesting from a clinical perspective in that it suggests that behaving in line with normatively salient value content may have some relationship with well-being. It also adds nuance to previously reported congruence effects between personal and culturally salient values. Our findings may suggest that it is not so much what individuals personally endorse that is important, but whether they feel that they have acted in line with socially or culturally salient values that may influence their well-being. An interesting further observation is that even people in the control condition, on average, felt as though they should have done more of the neutral behaviors (the means for perceived behavioral gaps were significantly above zero). The selected behaviors may have still tapped into salient motives and values; for example, “How many times have you chatted to someone before a lecture in the last 7 days?” could be seen as desirable behavior which increases social connectedness (e.g., self-transcendence value-expressive behavior). During our planning stages, we found that it is difficult to identify motivationally neutral behaviors. People may also have a bias toward believing they should have done more of any behavior even when the behavior is relatively neutral [as often indicted by a preference for action over inaction, see McCulloch et al. (2012)]. It is noteworthy that the currently used value-behavior scales have not been tested for social desirability yet. Furthermore, we currently have little objective information about behavior base rates beyond self-report rating scales on scales that already confound opportunities with frequencies. One option for future studies is to pre-determine the desirability of the behaviors in specific populations and use more objectively-informed base rates for behavioral frequencies.

Our second hypothesis was that if perceived discrepancies between ideal and reported value-expressive behaviors can reduce well-being, then participants in the experimental conditions should experience less well-being compared to controls who did not experience such discrepancies. Our results showed that being in an experimental group compared to a control group was not associated with more Negative Affect. We found a small effect size, which was not significant indicating slightly lower Positive Well-being in the experimental compared to the control condition. Individuals in the experimental condition reported greater search for meaning in life (which was not mediated by perceived behavioral gap). During the review process, a reviewer commented that our method does not allow testing to what extent any effects on well-being are related to making value content salient, to perceived incongruences with behavior, or an interaction of these two effects because the control condition differed in both value content and whether the scales were stretched or not. However, we think two observations are worth noting. First, the perceived behavioral gaps were comparable between the pooled value conditions group and control group despite these differences (see footnote 2). Second, Meaning in Life Searching was not significantly correlated with perceived behavioral gap. Because of these patterns, we conclude that the difference in Meaning in Life Searching between being in a value condition or not was likely dependent on the value content but not perceived behavioral gaps. In our exploratory analysis comparing each condition to the control across the three well-being measures, we did not find any significant differences. However, Meaning in Life Searching trended toward being highest for those in the self-enhancement condition compared to the others. This further lends support to the idea that value content contributed to the differences seen in Meaning in Life Searching, as self-enhancement differed to the other value conditions only in value content. We conclude that being asked about value-expressive behaviors in our study was associated with greater Meaning in Life Searching, particularly when asked about self-enhancement. This may be because the acts of thinking about value-expressive behaviors and why they are important, especially behaviors that participants did not feel were culturally important, induced greater levels of reflection on specific value-expressive behaviors and therefore resulted in greater scores of Meaning in Life Searching compared to controls.

In support of our third hypothesis, the perceived behavioral gaps in engaging in value-expressive behaviors were associated with lower Positive Well-being and greater Negative Affect. This suggests overall that not behaving in a way consistent with values (or inaction in general) is detrimental to our well-being. While the relationship between behavioral gap and Meaning in Life Searching was in the predicted direction, it was not significant. It is notable that although this study used only a brief manipulation to elicit relatively small perceived behavioral gaps, these perceived gaps still predicted negative well-being outcomes. The sizes of these effects were relatively small (explaining 1–3% of the variance), however in real-world situations, someone's behavior may be consistently and more dramatically incongruent with their values. Since we found an effect of perceived behavioral gap across all conditions, our results would predict that this perceived discrepancy would have a negative impact on their well-being in general. Our study implies that lessening the perceived behavioral gap by acting congruently with one's expectations of oneself should improve well-being, as the ACT model (Hayes et al., 1999) and Vowles and McCracken's (2008) study with chronic pain patients suggest. The only variable for which we did not find a perceived behavioral gap effect was Meaning in Life Searching. At the same time, the only variable affected by being in a values condition was Meaning in Life Searching. It is possible that the different aspects of our study—the failure to live up to expectations and being asked about values—affected different types of well-being such as subjective and psychological, or hedonic and eudaimonic, differently. Future studies could investigate this difference by having outcome measures that clearly discriminate between types of well-being.

Concerning our fourth hypothesis that our manipulation would result in poorer well-being, and that this would be mediated by behavioral gap, we did not find supporting evidence for any of our well-being outcomes. Instead, perceived behavioral gap alone was associated with Positive Well-being and Negative Affect, and experimental condition alone led to more Meaning in Life Searching. In addition to the points outlined above, it is possible that each experimental condition had different relationships to perceived behavioral gaps and well-being outcomes and that these canceled each other out when all experimental conditions were combined for the mediation analysis. For example, the self-enhancement condition led to less behavioral gap than the control, while self-transcendence led to more. Although we combined the experimental conditions to avoid the rate of error associated with what would have otherwise been 12 different mediation analyses, doing so may have masked the presence of mediations for some of our experimental value conditions. We conducted an exploratory analysis to examine whether manipulation of value-expressive behavior (self-enhancement, self-transcendence, conservation, and openness to change) compared to the control condition affected the three well-being components of Positive Well-being, Negative Affect, and Meaning in Life Searching. The main effect of value condition on measures of well-being was non-significant, showing no differential effect of experimental conditions on well-being. This seems to lend some support to clinical work that emphasizes that value content is less relevant for well-being as long as the value is of importance to the individual, but it does not fit with social psychological research (e.g., Kasser and Ryan, 1996; Ryan and Deci, 2000). Given the findings in social psychology research, it may be possible that our expected effect size of *f* = 0.30 used in our power analysis was an overestimation, considering that previous research has found the direct effect of values to be relatively small (e.g., Sagiv and Schwartz, 2000). For example, we found a trend showing that the manipulation of self-enhancement expressive behaviors increased Meaning in Life Searching. Other weak effects might be present, but our study may not have had enough power to pick up relatively weak value-specific effects. This could be addressed in future research with larger sample sizes.

Concerning our final hypothesis, we also hypothesized that personal values and manipulation of value-expressive behavior would interact to produce greater effects on behavior gap when personal values and manipulated value-expressive behaviors were complementary or aligned. Values are often seen as strong motivational guides of behavior and therefore, we expected a situational manipulation of a behavior that is aligned with a value that someone finds personally important to have a greater effect than a manipulation of value-expressive behaviors that a person does not personally prioritize. However, there was no evidence for this mechanism and the effects on perceived behavioral gap were independent of personal value priorities. As we discussed above, it suggests that normatively salient values may drive well-being effects in a healthy population. Overall, our findings suggest that value content matters, but it is important to distinguish between personally important values and socially salient values.

A limitation of our study is how the experimental conditions compared to controls differed in two ways simultaneously: following the previous paradigm developed by Yousaf and Gobet (2013), participants had to first provide reasons for the importance of a particular value-expressive behavior (phase 1) followed by rating each behavior on manipulated response scales (phase 2). Therefore, we do not know whether an increase in behavior gap was due to making the behaviors salient, the manipulated response scales, or both. However, the fact that the control group experienced perceptions of behavioral gaps similar to those in the combined values group suggests that behavioral gaps can be induced even without artificially stretching response scales. Instead, the differences seen between these two groups in Meaning in Life Searching seem to be more dependent on value content. Future studies are needed to separate the two effects experimentally to identify which mechanism is more important for producing effects on well-being. For example, a more complex design could cross the two conditions (e.g., one condition only includes the salience manipulation, whereas the other condition only includes the stretched response scales to induce perceived behavioral gaps). Our study has demonstrated that the method has some promise and that motivational content of values seems to have an effect. Future studies can start disentangling these mechanisms further.

Further improvements could be made to the method used in our study. For example, phase 1 could relate values directly to the participant by asking how important each of the behaviors are for the participant. Alternatively, a researcher could ask what behaviors express the respondent's own values. This could help ensure the manipulation relates to personal values rather than implied social norms. Second, in phase 3, participants could be asked how they think they should have acted considering their own values. Currently, the instruction could be interpreted as asking how participants should have acted taking into consideration how others behave, which may activate a sense of social norms instead of personal values. Last, discrepancy between values and actual behavior could be induced in different ways, for example, by preventing helping behavior in an interaction for participants high in self-transcendence.

In summary, we investigated the relationship between values, behavior, and well-being, drawing upon diverse theoretical perspectives and using a novel experimental manipulation. The manipulation worked as intended for the self-transcendence and openness to change related behaviors—these manipulations increased perceived behavioral gap compared to the control group. Our findings also suggest that future studies need to disentangle personal value preferences from normative salience, as implied by our complex findings. Therefore, our method shows promise and could be refined and used in future value-behavior studies. It may be particularly interesting to use variations of this procedure to tease apart possible differential effects of values on behaviors in different contexts. For example, could not behaving on self-enhancement values lead to greater perceived behavioral gaps in achievement relevant contexts or among more career-oriented populations? At the same time, our study has demonstrated that perceived behavioral gaps (perceptions of not acting) can be detrimental to well-being overall. In making these connections, we are the first to conceptually link Schwartz' value theory to clinical models such as ACT (Hayes et al., 1999). There is much potential for cross-fertilization: clinical models that identify value-behavior congruence as a key factor in creating a meaningful life and reducing emotional distress could incorporate the different motivational goals as described by Schwartz. Similarly, clinical research may provide novel insights into how values play out in the day-to-day experience of people.

## Ethics Statement

This study was carried out in accordance with the recommendations of Human Ethics Guidelines, Victoria University of Wellington Human Ethics Committee. The protocol was approved by the School of Psychology Human Ethics Committee under delegated authority of Victoria University of Wellington's Human Ethics Committee. All subjects gave written informed consent in accordance with the Declaration of Helsinki.

## Author Contributions

All authors contributed to the design of the study. MC and JK completed the data collection and analysis. MC. drafted the manuscript. All authors contributed to editing the manuscript for publication. RF supervised this research.

### Conflict of Interest Statement

The authors declare that the research was conducted in the absence of any commercial or financial relationships that could be construed as a potential conflict of interest.

## Footnotes

^1^Keyes et al. (2002) differentiate between subjective well-being and psychological well-being; the former describes the presence of positive affect over negative affect and positive evaluations on one's life, while the latter describes a positive perception of the self and having a meaningful life. Others differentiate between hedonic and eudaimonic well-being (e.g., Huta, 2013). The current study uses measures of many types of well-being and will henceforth refer simply to well-being as a general construct encompassing these different aspects.

^2^A Welch's *t*-test was conducted to see if the difference between the behavioral gaps for the combined values conditions (*M* = 0.82, *SD* = 0.88) and control (*M* = 0.58, *SD* = 0.72) were significantly different and they were not, *t*_(51.24)_ = −1.57, *p* = 0.12, *d* = 0.28.

^3^Because the manipulation check suggested that the manipulation did not work as intended for the conservation and self-enhancement value conditions, we repeated the MANOVA with these conditions removed. Results from this analysis were non-significant, Pillais' Trace = 0.07, *F*_(6, 178)_ = 1.00, *p* = 0.43, indicating no main effect of experimentally manipulated values condition on measures of well-being when conservation and self-enhancement were excluded.
