# Supplementary material for: The Complexities of “Minding the Gap”: Perceived Discrepancies Between Values and Behavior Affect Well-Being
Source: Front Psychol. 2019 Apr 9;10:736. doi: 10.3389/fpsyg.2019.00736 (PMC6465641; doi:10.3389/fpsyg.2019.00736)
Supplement: Supplementary file 1 [file Data_Sheet_1.pdf]

Appendix A

Phase One Questions

Please finish the statements below with a reason/reasons why these behaviours are important for people in general to do. You are required to write at least 140 characters for each behaviour.

Self-Transcendence. It is important for people to support friends and family with everyday tasks, such as baby-sitting, because…

It is important for people to buy environmentally friendly products because…

It is important for people to help out friends and classmates at university because…

It is important for people to donate money to charity because…

It is important for people to give money to homeless people because…

It is important to speak out in favour of tolerance for those with different lifestyles because…

Conservation. It is important for people to keep their opinions to themselves in order to not disagree with others because…

It is important for people not to boast about their own achievements because…

It is important for people to change their passwords regularly because…

It is important for people to check the expiration date on products before buying or using them because…

It is important for people to attend dawn services on ANZAC Day because...

It is important for people to wait for the green light before crossing the street, even when no cars are coming, because…

Self-Enhancement. It is important for people to copy the behaviour of rich people because…

It is important for people to influence others to get what they want because…

It is important for people to insist that others do what they want them to do because…

It is important for people to show their wealth because…

It is important for people to study hard in order to get a good grade in a difficult course because...

It is important for people to try to impress their tutor/lecturer by doing more work than is expected of them because...

Openness to Change. It is important for people to act independently without consulting other people because…

It is important for people to change their routine regularly because…

It is important for people to treat themselves because…

It is important for people to learn something simply for the joy of learning because…

It is important for people to be spontaneous because…

It is important for people to keep informed on topics not related to their studies or profession because…

Phase Two Questions

You will now be asked about your past engagement in certain behaviours. Please answer truthfully.

Self-Transcendence. How much time have you spent supporting friends and family with everyday tasks, such as baby-sitting, in the last 7 days?

0 minutes	1-30 minutes	More than 30 minutes	More than 1 hour	More than 2 hours	More than 4 hours	More than 7 hours	More than 12 hours	More than 20 hours

How many times have you bought environmentally friendly products in the last 7 days?

None at all	1 time	2 times	3-5 times	6-8 times	9-11 times	12-14 times	15-17 times	18+ times

How many times have you helped out friends and classmates at university in the last 7 days?

None at all	1 time	2 times	3-5 times	6-8 times	9-11 times	12-14 times	15-17 times	18+ times

How many times have you spoken out in favour of tolerance for those with different lifestyles in the last 7 days?

None at all	1 time	2 times	3-5 times	6-8 times	9-11 times	12-14 times	15-17 times	18+ times

How much money have you donated to charity in the last month?

$0	10c-$1	More than $1	More than $10	More than $20	More than $50	More than $80	More than $110	More than $150

How much money have you given to homeless people in the last month?

$0	10c-$1	More than $1	More than $10	More than $20	More than $50	More than $80	More than $110	More than $150

Conservation. How many times did you keep your opinion to yourself in order to not disagree with others yesterday?

None at all	1 time	2 times	3-5 times	6-8 times	9-11 times	12-14 times	15-17 times	18+ times

How many times have you refrained from telling someone about your achievements in the last month?

None at all	1 time	2 times	3-5 times	6-8 times	9-11 times	12-14 times	15-17 times	18+ times

How many times have you changed your passwords in the last year?

None at all	1 time	2 times	3-5 times	6-8 times	9-11 times	12-14 times	15-17 times	18+ times

How many times have you checked the expiration date on products before buying or using them in the last 7 days?

None at all	1 time	2 times	3-5 times	6-8 times	9-11 times	12-14 times	15-17 times	18+ times

How many times have you attended a dawn service for ANZAC Day in the last 10 years?

None at all	1 time	2 times	3 times	4 times	5 times	6 times	7-8 times	9-10 times

How many times have you waited for the green light before crossing the street, even when no cars were coming, in the last 3 days?

None at all	1-3 times	4-6 times	7-10 times	11-14 times	15-19 times	20-24 times	25-29 times	30+ times

Self-Enhancement. How many times have you tried to impress your tutor/lecturer by taking on more work this year?

None at all	1 time	2 times	3-5 times	6-8 times	9-11 times	12-14 times	15-17 times	18+ times

How many times have you copied the behaviour of rich people in the last 7 days?

None at all	1 time	2 times	3-5 times	6-8 times	9-11 times	12-14 times	15-17 times	18+ times

How many times have you influenced others to get what you want in the last 7 days?

None at all	1 time	2 times	3-5 times	6-8 times	9-11 times	12-14 times	15-17 times	18+ times

How many times have you insisted that others do what you want them to do in the last 7 days?

None at all	1 time	2 times	3-5 times	6-8 times	9-11 times	12-14 times	15-17 times	18+ times

How many times have you shown your wealth in the last 7 days?

None at all	1 time	2 times	3-5 times	6-8 times	9-11 times	12-14 times	15-17 times	18+ times

How much time have you spent studying in order to get a good grade in a difficult course in the last 7 days?

0 minutes	1-30 minutes	More than 30 minutes	More than 1 hour	More than 2 hours	More than 4 hours	More than 7 hours	More than 12 hours	More than 20 hours

Openness to Change. How many times have you acted independently without consulting other people in the last 7 days?

None at all	1 time	2 times	3-5 times	6-8 times	9-11 times	12-14 times	15-17 times	18+ times

How many times have you changed your routine in the last month?

None at all	1 time	2 times	3-5 times	6-8 times	9-11 times	12-14 times	15-17 times	18+ times

How many times have you learnt something simply for the joy of learning in the last 7 days?

None at all	1 time	2 times	3-5 times	6-8 times	9-11 times	12-14 times	15-17 times	18+ times

How many times have you treated yourself in the last 7 days?

None at all	1 time	2 times	3-5 times	6-8 times	9-11 times	12-14 times	15-17 times	18+ times

How many times have you been spontaneous in the last 7 days?

None at all	1 time	2 times	3-5 times	6-8 times	9-11 times	12-14 times	15-17 times	18+ times

How many times have you kept informed on topics not related to your studies or profession in the last 7 days?

None at all	1 time	2 times	3-5 times	6-8 times	9-11 times	12-14 times	15-17 times	18+ times

Control. How many times have you chatted to someone before a lecture in the last 7 days?

None at all	1-2 times	3-4 times	5-6 times	7-8 times	9-10 times	11-12 times	13-14 times	15+ times

How many times have you Googled something in the last 3 days?

None at all	1-5 times	6-10 times	11-20 times	21-30 times	31-40 times	41-50 times	51-60 times	61+ times

How many times have you used your phone to call someone or answer a call in the last 7 days?

None at all	1-2 times	3-4 times	5-6 times	7-8 times	9-10 times	11-12 times	13-14 times	15+ times

How many times have you used the Info Ihonui in the last month?

None at all	1 time	2 times	3 times	4 times	5 times	6 times	7 times	8+ times

How many times have you used a computer at one of the VUW libraries in the last 7 days?

None at all	1-2 times	3-4 times	5-6 times	7-8 times	9-10 times	11-12 times	13-14 times	15+ times

How many times have you visited a news website in the last 7 days?

None at all	1-3 times	4-6 times	7-9 times	10-12 times	13-15 times	16-18 times	19-21 times	22+ times

Phase Three Questions

You will now be asked about how you think you ***should*** have behaved in the past. Please answer truthfully.

Self-Transcendence. In the last 7 days, how many times do you think you ***should*** have bought environmentally friendly products?

A lot less often than I did -4	-3	-2	-1	The same amount that I did 0	1	2	3	A lot more often than I did 4

In the last 7 days, how many times do you think you ***should*** have helped out friends and classmates at university?

A lot less often than I did -4	-3	-2	-1	The same amount that I did 0	1	2	3	A lot more often than I did 4

In the last 7 days, how many times do you think you ***should*** have bought environmentally friendly products?

A lot less often than I did -4	-3	-2	-1	The same amount that I did 0	1	2	3	A lot more often than I did 4

In the last 7 days, how many times do you think you ***should*** have spoken out in favour of tolerance for those with different lifestyles?

A lot less often than I did -4	-3	-2	-1	The same amount that I did 0	1	2	3	A lot more often than I did 4

In the past month, how much money do you think you ***should*** have donated to charity?

A lot less money than I did -4	-3	-2	-1	The same amount of money that I did 0	1	2	3	A lot more money than I did 4

In the past month, how much money do you think you ***should*** have given to homeless people?

A lot less money than I did -4	-3	-2	-1	The same amount of money that I did 0	1	2	3	A lot more money than I did 4

Conservation. How many times do you think you ***should*** have kept your opinion to yourself in order to not disagree with others yesterday?

A lot less often than I did -4	-3	-2	-1	The same amount that I did 0	1	2	3	A lot more often than I did 4

In the past month, how many times do you think you ***should*** have refrained from telling someone about your achievements?

A lot less often	-3	-2	-1	The same amount	1	2	3	A lot more often

than I did -4	that I did 0	than I did 4

In the last year, how many times do you think you ***should*** have changed your passwords?

A lot less often than I did -4	-3	-2	-1	The same amount that I did 0	1	2	3	A lot more often than I did 4

In the last 7 days, how many times do you think you ***should*** have checked the expiration date on products before buying or using them?

A lot less often than I did -4	-3	-2	-1	The same amount that I did 0	1	2	3	A lot more often than I did 4

In the last 10 years, how many times do you think you ***should*** have attended dawn services on ANZAC Day?

A lot less often than I did -4	-3	-2	-1	The same amount that I did 0	1	2	3	A lot more often than I did 4

Self-Enhancement. In the past month, how many times do you think you ***should*** have tried to impress your tutor/lecturer by taking on more work?

A lot less often than I did -4	-3	-2	-1	The same amount that I did 0	1	2	3	A lot more often than I did 4

In the last 7 days, how many times do you think you ***should*** have copied the behaviour of rich people?

A lot less often	-3	-2	-1	The same amount	1	2	3	A lot more often

than I did -4				that I did 0				than I did 4

In the last 7 days, how many times do you think you ***should*** have influenced others to get what you wanted?

A lot less often than I did -4	-3	-2	-1	The same amount that I did 0	1	2	3	A lot more often than I did 4

In the last 7 days, how many times do you think you ***should*** have insisted that others do what you wanted them to do?

A lot less often than I did -4	-3	-2	-1	The same amount that I did 0	1	2	3	A lot more often than I did 4

In the last 7 days, how many times do you think you ***should*** have shown your wealth?

A lot less often than I did -4	-3	-2	-1	The same amount that I did 0	1	2	3	A lot more often than I did 4

In the last 7 days, how much time do you think you ***should*** have spent studying in order to get a good grade in a difficult test/assignment?

A lot less often than I did -4	-3	-2	-1	The same amount that I did 0	1	2	3	A lot more often than I did 4

Openness to Change. In the last 7 days, how many times do you think you ***should*** have acted independently without consulting other people?

A lot less often than I did	-3	-2	-1	The same amount that I did	1	2	3	A lot more often than I did

-4				0				4

In the past month, how many times do you think you ***should*** have changed your routine?

A lot less often than I did -4	-3	-2	-1	The same amount that I did 0	1	2	3	A lot more often than I did 4

In the last 7 days, how many times do you think you ***should*** have learnt something simply for the joy of learning?

A lot less often than I did -4	-3	-2	-1	The same amount that I did 0	1	2	3	A lot more often than I did 4

In the last 7 days, how many times do you think you ***should*** have treated yourself?

A lot less often than I did -4	-3	-2	-1	The same amount that I did 0	1	2	3	A lot more often than I did 4

In the last 7 days, how many times do you think you ***should*** have been spontaneous?

A lot less often than I did -4	-3	-2	-1	The same amount that I did 0	1	2	3	A lot more often than I did 4

In the last 7 days, how many times do you think you ***should*** have kept informed on topics not related to your studies or profession?

A lot less often than I did -4	-3	-2	-1	The same amount that I did 0	1	2	3	A lot more often than I did 4

Control. How many times do you think you ***should*** have chatted to someone before a lecture in the last 7 days?

A lot less often than I did	-3	-2	-1	The same amount that I did	1	2	3	A lot more often than I did
-4				0				4

How many times do you think you ***should*** have Googled something in the last 3 days?

A lot less often than I did	-3	-2	-1	The same amount that I did	1	2	3	A lot more often than I did
-4				0				4

How many times do you think you ***should*** have used your phone to call someone or answer a call in the last 7 days?

A lot less often than I did	-3	-2	-1	The same amount that I did	1	2	3	A lot more often than I did
-4				0				4

How many times do you think you ***should*** have used the Info Ihonui in the last month?

A lot less often than I did	-3	-2	-1	The same amount that I did	1	2	3	A lot more often than I did
-4				0				4

How many times do you think you ***should*** have used a computer at one of the VUW libraries in the last 7 days?

A lot less often than I did	-3	-2	-1	The same amount that I did	1	2	3	A lot more often than I did
-4				0				4

How many times do you think you ***should*** have visited a news website in the last 7 days?

A lot less often than I did	-3	-2	-1	The same amount that I did	1	2	3	A lot more often than I did
-4				0				4

Appendix B

Table A1. *Task descriptions and examples for each survey component for each condition*

	Control	Self-Enhancement	Self-Transcendence	Conservation	Openness to Change
Personal values			50% of all participants complete PVQ-RR first		
Phase 1 Sentence completion description	Respond to 6 sentence prompts for random selection of behaviors from each value condition.	Respond to 6 sentence prompts for behaviors expressive of self-enhancement.	Respond to 6 sentence prompts for behaviors expressive of self-transcendence	Respond to 6 sentence prompts for behaviors expressive of conservation	Respond to 6 sentence prompts for behaviors expressive of openness to change
Phase 1 Example item	"It is important for people to show their wealth because…"	"It is important for people to show their wealth because…"	"It is important for people to donate money to charity because…"	"It is important for people to change their passwords regularly because…"	"It is important for people to treat themselves because…"
Phase 2 Past behaviour description	Report extent of completed neutral behaviors on unstretched scales	Report extent of completed behaviors expressive of self-enhancement on stretched scales	Report extent of completed behaviors expressive of self-transcendence on stretched scales	Report extent of completed behaviors expressive of conservation on stretched scales	Report extent of completed behaviors expressive of openness to change on stretched scales
Phase 2 Example item	"How many times have you Googled something in the last 3 days?"	"How many times have you shown your wealth in the last 7 days?"	"How much money have you donated to charity in the last month?"	"How many times have you changed your passwords in the last year?"	"How many times have you treated yourself in the last 7 days?"
Phase 3 Behavioral gap description	Participants report how much they think they should have completed behaviors asked about previously	Participants report how much they think they should have completed behaviors asked about previously	Participants report how much they think they should have completed behaviors asked about previously	Participants report how much they think they should have completed behaviors asked about previously	Participants report how much they think they should have completed behaviors asked about previously
Phase 3 Example item	"How many times do you think you ***should*** have Googled something in the last 3 days?"	"In the last 7 days, how many times do you think you ***should*** have shown your wealth?"	"In the past month, how much money do you think you ***should*** have donated to charity?"	"In the last year, how many times do you think you ***should*** have changed your passwords?"	"In the last 7 days, how many times do you think you ***should*** have treated yourself?"
Well-being measures		All participants complete measures of various aspects of well-being.			
Personal values			50% of all participants complete PVQ-RR last		

Appendix C

Shortened PVQ

Here we briefly describe different people. Please read each description and think about how much that person is or is not like you. Choose an answer that shows how much the person described is like you. [Items are rated from *Not like me at all, Not like me, A little like me, Moderately like me, Like me, Very much like me*]

It is important to them to be free to choose what they do by themselves. [OtC]

It is important to them that everyone be treated justly, even people they don't know. [ST]

It is important to them to have all sorts of new experiences. [OtC]

It is important to them to be the one who tells others what to do. [SE]

It is important to them to figure things out themselves. [OtC]

It is important to them to enjoy life's pleasures. [OtC]

It is important to them to follow rules even when no-one is watching. [CONS]

It is important to them to be very successful. [SE]

It is very important to them to help the people dear to them. [ST]

It is important to them to be personally safe and secure. [CONS]

It is important to them to be a dependable and trustworthy friend. [ST]

It is important to them to be wealthy. [SE]

It is important to them to maintain traditional values and ways of thinking. [CONS]

It is important to them to be tolerant toward all kinds of people and groups. [ST]

It is important to them to care for nature. [ST]

It is important to them to avoid upsetting other people. [CONS]

It is important to them that their country is secure and stable. [CONS]

It is important to them to be humble. [CONS]

It is important to them to protect their public image. [CONS]

196

Appendix D

Table A2

The Main and Interaction Effects of Condition and Personal Values on Behavioral Gap

	Estimate	Standard error	T value	P value
(Intercept)	.33	1.23	.27	.79
Condition CONS	1.52	1.67	.91	.37
Condition SE	-1.10	1.84	-.60	.55
Condition ST	-3.01	2.09	-1.44	.15
Condition OTC	-.16	1.72	-.10	.93
PVQ-RR CONS	-.05	.19	-.26	.80
PVQ-RR OTC	-.08	.23	-.33	.74
PVQ-RR SE	-.07	.15	-.45	.65
PVQ-RR ST	.20	.27	.74	.46
Condition CONS: PVQ-RR CONS	-.13	.29	-.43	.67
Condition SE: PVQ-RR CONS	.04	.33	.11	.91
Condition ST: PVQ-RR CONS	.28	.31	.89	.37
Condition OTC: PVQ-RR CONS	.04	.40	.11	.92
Condition CONS: PVQ-RR OTC	-.08	.35	-.24	.81
Condition SE: PVQ-RR OTC	-.18	.33	-.55	.59
Condition ST: PVQ-RR OTC	-.03	.36	-.08	.94
Condition OTC: PVQ-RR OTC	.16	.37	.42	.67
Condition CONS: PVQ-RR SE	.22	.22	.99	.32
Condition SE: PVQ-RR SE	.13	.24	.56	.58
Condition ST: PVQ-RR SE	.23	.25	.90	.37
Condition OTC: PVQ-RR SE	.44	.30	1.46	.15
Condition CONS: PVQ-RR ST	-.20	.38	-.53	.60
Condition SE: PVQ-RR ST	.18	.43	.43	.67
Condition ST: PVQ-RR ST	.32	.43	.76	.45
Condition OTC: PVQ-RR ST	-.35	.48	-.74	.46

Note. CONS = conservation, SE = self-enhancement, ST = self-transcendence, OTC = openness to change

Table A3

The Main and Interaction Effects of Condition and Personal Values on Positive Well-Being

	Estimate	Standard error	T value	P value
(Intercept)	2.26	1.04	2.18	.03*
Condition CONS	2.24	1.41	1.60	.11
Condition SE	.30	1.56	.20	.85
Condition ST	.71	1.77	.40	.69
Condition OTC	-1.08	1.50	-.74	.46
PVQ-RR CONS	-.01	.16	-.05	.96
PVQ-RR OTC	.12	.19	.60	.55
PVQ-RR SE	-.04	.13	-.31	.76
PVQ-RR ST	.16	.23	.67	.50
Condition CONS: PVQ-RR CONS	-.06	.24	-.25	.81
Condition SE: PVQ-RR CONS	.14	.28	.49	.63
Condition ST: PVQ-RR CONS	-.09	.26	-.35	.73
Condition OTC: PVQ-RR CONS	-.14	.34	-.41	.68
Condition CONS: PVQ-RR OTC	-.14	.30	-.47	.64
Condition SE: PVQ-RR OTC	-.10	.28	-.37	.71
Condition ST: PVQ-RR OTC	-.02	.30	-.06	.96
Condition OTC: PVQ-RR OTC	.21	.32	.66	.51
Condition CONS: PVQ-RR SE	.12	.19	.66	.51
Condition SE: PVQ-RR SE	.06	.20	.30	.77
Condition ST: PVQ-RR SE	-.10	.22	-.46	.64
Condition OTC: PVQ-RR SE	.18	.26	.71	.48
Condition CONS: PVQ-RR ST	-.40	.32	-1.24	.22
Condition SE: PVQ-RR ST	-.14	.37	-.37	.71
Condition ST: PVQ-RR ST	.04	.36	.11	.92
Condition OTC: PVQ-RR ST	-.01	.41	-.02	.99

Note. CONS = conservation, SE = self-enhancement, ST = self-transcendence, OTC = openness to change

Table A4

The Main and Interaction Effects of Condition and Personal Values on Meaning in Life Searching

	Estimate	Standard error	T value	P value
(Intercept)	3.75	1.83	2.05	.04*
Condition CONS	-2.47	2.48	-1.00	.32
Condition SE	-.86	2.74	-.31	.75
Condition ST	1.62	3.11	.52	.60
Condition OTC	-2.23	2.56	-.87	.38
PVQ-RR CONS	.15	.28	.52	.60
PVQ-RR OTC	-.65	.34	-1.89	.06
PVQ-RR SE	.27	.22	1.20	0.23
PVQ-RR ST	.35	.41	.85	.40
Condition CONS: PVQ-RR CONS	.31	.43	.73	.47
Condition SE: PVQ-RR CONS	.20	.49	.40	.69
Condition ST: PVQ-RR CONS	.09	.46	.19	.85
Condition OTC: PVQ-RR CONS	.52	.60	.88	.38
Condition CONS: PVQ-RR OTC	.87	.52	1.67	.10
Condition SE: PVQ-RR OTC	.89	.49	1.80	.07
Condition ST: PVQ-RR OTC	.78	.53	1.45	.15
Condition OTC: PVQ-RR OTC	.75	.56	1.36	.18
Condition CONS: PVQ-RR SE	.33	.33	1.00	.32
Condition SE: PVQ-RR SE	-.21	.36	-.56	.55
Condition ST: PVQ-RR SE	-.21	.38	-.55	.58
Condition OTC: PVQ-RR SE	.37	.45	.83	.41
Condition CONS: PVQ-RR ST	-.71	.57	-1.25	.21
Condition SE: PVQ-RR ST	-.48	.64	-.75	.45
Condition ST: PVQ-RR ST	-.86	.63	-1.36	.18
Condition OTC: PVQ-RR ST	-.84	.71	-1.19	.24

Note. CONS = conservation, SE = self-enhancement, ST = self-transcendence, OTC = openness to change

Table A5

The Main and Interaction Effects of Condition and Personal Values on Negative Affect

	Estimate	Standard error	T value	P value
(Intercept)	.65	1.07	.60	.55
Condition CONS	2.90	1.46	2.00	.05*
Condition SE	2.05	1.61	1.28	.20
Condition ST	2.77	1.83	1.52	.13
Condition OTC	2.09	1.50	1.39	.17
PVQ-RR CONS	.09	.16	.53	.60
PVQ-RR OTC	.003	.20	.02	.99
PVQ-RR SE	.01	.13	.05	.96
PVQ-RR ST	.14	.24	.57	.57
Condition CONS: PVQ-RR CONS	-.03	.25	-.12	.91
Condition SE: PVQ-RR CONS	-.49	.29	-1.69	.09
Condition ST: PVQ-RR CONS	-.25	.27	-.91	.36
Condition OTC: PVQ-RR CONS	.24	.35	.68	.50
Condition CONS: PVQ-RR OTC	-.55	.31	-1.80	.07
Condition SE: PVQ-RR OTC	-.04	.29	-.15	.89
Condition ST: PVQ-RR OTC	-.02	.31	-.05	.96
Condition OTC: PVQ-RR OTC	-.18	.33	-.56	.58
Condition CONS: PVQ-RR SE	.05	.19	.28	.78
Condition SE: PVQ-RR SE	.05	.21	.23	.82
Condition ST: PVQ-RR SE	.11	.22	.50	.62
Condition OTC: PVQ-RR SE	-.01	.26	-.04	.97
Condition CONS: PVQ-RR ST	-.04	.33	-.13	.90
Condition SE: PVQ-RR ST	-.01	.38	-.03	.98
Condition ST: PVQ-RR ST	-.40	.37	-1.06	.29
Condition OTC: PVQ-RR ST	-.43	.42	-1.04	.30

Note. CONS = conservation, SE = self-enhancement, ST = self-transcendence, OTC = openness to change
